# Lessons Learned from the COVID-19 Pandemic in Nursing Homes: A Systematic Review

**DOI:** 10.3390/ijerph192416919

**Published:** 2022-12-16

**Authors:** Marina Martínez-Payá, Irene Carrillo, Mercedes Guilabert

**Affiliations:** Health Psychology Department, Miguel Hernandez University, 03202 Elche, Spain

**Keywords:** nursing homes, long-term care, residential facilities, COVID-19, pandemic

## Abstract

Nursing homes are one of the hardest-hit environments in terms of mortality from COVID-19. Given the reactive management of the pandemic, it is necessary to reflect on, and answer, the question as to which good practices (interventions) were implemented in care homes (population) to improve management and care quality (outcomes). This systematic review aimed to identify and describe good practices adopted in care homes during the COVID-19 pandemic or other recent epidemics. We conducted searches in Embase, PubMed, ScienceDirect, ProQuest Central, and Scopus over the period 1–30 November, 2021, using the descriptors “nursing homes”, “long-term care”, “long-term care facilities” and “COVID-19”; and the keywords “learnings”, “lessons”, “positive learnings”, “positive lessons”, “SARS”, “MERS”, “COVID-19” and “pandemic”. We identified 15 papers describing 14 best practices and 26 specific actions taken for COVID-19 management in long-term care facilities. Following the IDEF methodology, the practices were classified into strategic processes (staff training, communication with the national health system, person-centered care, and protocols), operational processes (cohorts, diagnostic testing, case monitoring, personal protective equipment, staff reinforcement, restriction of visits, social distancing, and alternative means for communication with families) and support processes (provision of equipment and hygiene reinforcement). Fifty percent of practices were likely to be maintained beyond the outbreak to improve the operation and quality of the long-term care facilities. This review summarizes the most common measures adopted to manage the COVID-19 pandemic in the context of increased vulnerability and highlights the deficiencies that must be addressed.

## 1. Introduction

The rapid spread of severe acute respiratory syndrome coronavirus 2 (SARS-CoV-2), which causes coronavirus disease 2019 (COVID-19), constitutes a global public health problem, due to the high incidence and mortality of the disease [1]. On 11 March 2020, the World Health Organization (WHO) characterized the COVID-19 outbreak as a pandemic. While anyone can contract the disease, it does not affect everyone equally. In most Western European countries, people aged under 60 years accounted for only 5% of people who died from the disease in 2020, while, in the USA, 21% of COVID deaths in 2020 occurred in people aged under 65 years [2]. Older adults are particularly vulnerable to COVID-19, as they tend to present more severe forms of the disease and have a worse prognosis due to comorbidity, geriatric syndromes and the frailty associated with ageing. For this reason, the COVID-19 pandemic was also considered a geriatric emergency [3,4,5]. Care homes constitute one of the hardest hit settings in terms of mortality, accounting for between 40% and 80% of COVID-19 deaths in the first six months of the pandemic [6]. This greater vulnerability is related to characteristics of care home residents that are risk factors for infection (age, comorbidities, specific care needs) and characteristics of the care homes themselves that facilitate the spread of infection (closed systems, proximity between users and staff) [7]. The close interaction between people in care homes increases the risk of death in institutionalized older adults compared with people who have the same age-related vulnerabilities but who live in more isolated settings, such as private homes [8].

This situation forced care homes to rapidly adapt their management strategies and care practices to curb the spread of the virus. It is crucial to identify these organizational, structural, and care-related modifications, and reflect on their justification and impact, to extract lessons that can be applied in future outbreaks or pandemics, or that can be maintained if they represent an improvement in terms of the pre-pandemic situation. To the best of our knowledge, no previous systematic reviews have evaluated lessons learned from the COVID-19 pandemic, or indeed from other recent epidemics (e.g., severe acute respiratory syndrome coronavirus 1 (SARS-CoV) in November 2002, in China [9], or Middle East respiratory syndrome coronavirus (MERS-CoV) in Saudi Arabia in April 2012, [10]) in the specific context of care homes.

The objective of this systematic review was to identify and describe good practices adopted in care homes during the COVID-19 pandemic or other recent epidemics. With our results, we intend to build a repository of actions that contribute to the continued improvement of care home functioning and that strengthen the ability of these facilities to deal with future health crises.

## 2. Materials and Methods

### 2.1. Design

We conducted this systematic review in accordance with the Preferred Reporting Items for Systematic Reviews and Meta-Analyses (PRISMA) statement [11]. The protocol for this review was approved and published in PROSPERO (international prospective register of systematic reviews) with the registration number CRD42021212172.

We formulated our research question using the PICo framework (population, phenomena of interest, context) [12], as follows: “Which good practices to improve management and care quality (phenomena of interest) have been implemented in care homes (population) during the COVID-19 pandemic (context)”?

### 2.2. Eligibility Criteria

We included studies that described or evaluated lessons learned from the COVID-19 pandemic, or from recent epidemics (SARS-CoV and MERS-CoV), in care homes for older people (aged over 65 years).

We defined good practices (or lessons learned) as any intervention adopted with the aim of adapting the care home to the pandemic situation or minimizing or curbing the spread of the virus. Eligible articles were published in English or Spanish between 2002 and 2021 and described practices adopted in residential care facilities for older adults. We restricted the search to articles published from 2002 because that was the year of the SARS-CoV outbreak in the southeast Chinese province of Guangdong. The first outbreak of MERS-CoV subsequently occurred in Saudi Arabia in April 2012. We included these years in our search because we considered the experience of these epidemics could establish a precedent in the application of good practices in care homes during health crises caused by the appearance and spread of highly infectious or lethal viruses.

We excluded publications that analyzed the effectiveness of vaccines or drugs for treating disease, reducing risk factors, or reducing the incidence of infections. We also excluded studies that described lessons learned in settings other than care homes, such as hospitals. The only publication type we excluded was letters to the editor.

One of the review authors was responsible for removing duplicates and screening the titles and abstracts to eliminate clearly ineligible publications. After retrieving the full-text articles of all potentially eligible records, two review authors independently assessed each one against the eligibility criteria, consulting a third review author in case of disagreement. We used Mendeley Reference Manager to simplify the selection process.

### 2.3. Sources of Information and Search Strategy

Between 1 and 30 November 2021, we conducted searches in Embase, PubMed, ScienceDirect, ProQuest Central and Scopus, using the descriptors “nursing homes”, “long-term care”, “long-term care facilities” and “COVID-19”; and the keywords “learnings”, “lessons”, “positive learnings”, “positive lessons”, “SARS”, “MERS”, “COVID-19” and “pandemic”. Table 1 presents the search strategies for each database and the number of articles returned.

### 2.4. Data Extraction and Analysis

First, from each included article, we extracted the title, first author, year of publication, objective, study design, sample size, description of practices or main findings, and conclusions; then we performed a narrative synthesis of these data (Table S1).

Second, we analyzed the lessons learned following the IDEFØ scheme (Integrated DEFinition Function Modeling Method), which models organizational processes as a series of interrelated functions [13]. Good practices were classified into three categories: operational processes (core processes developed in real time with residents), strategic processes (management processes that guided the execution of operational processes) and support processes (which provided resources or support to facilitate the other processes and provided coherence to the organization).

Lastly, we classified the good practices into the following five intervention categories: case and contact management, proactive detection, infection control, resource prioritization, and collaboration [14]. In this classification of good practices, we also recorded whether they were applied only in the outbreak situation or were maintained beyond the pandemic (because they helped to improve care home processes).

## 3. Results

### 3.1. Study Selection

**Figure 1:**
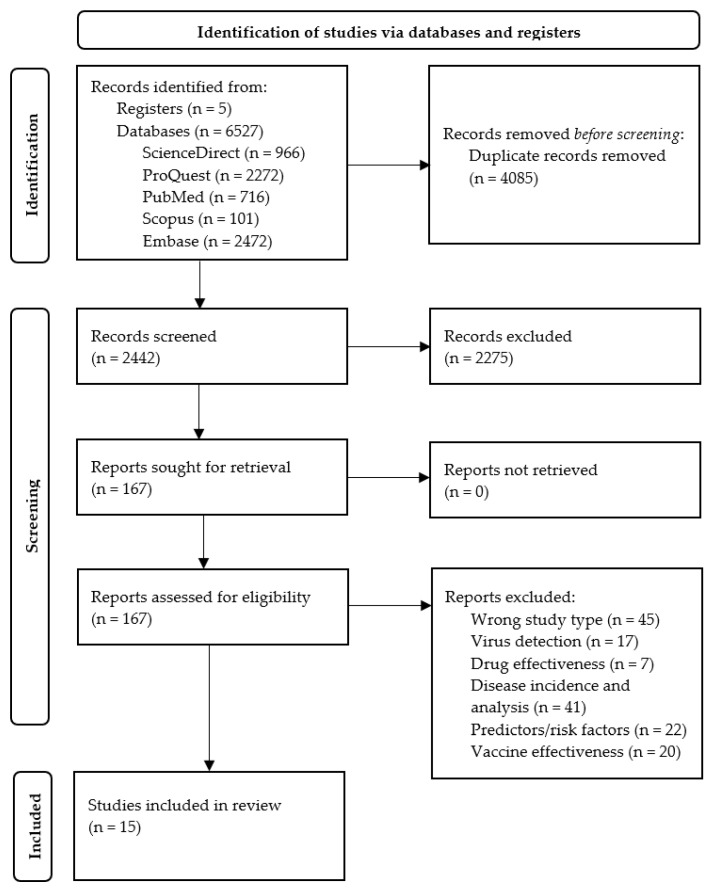
PRISMA 2020 flow diagram.

The electronic search recovered 6527 records. After removing 4085 duplicates, we screened 2442 records, of which we excluded 2275. Reasons for exclusion included: the study setting was in hospitals (specialized care, not long-term care facilities), adult or pediatric study population (rather than older adults), generic approach to pandemic management in gerontological facilities, no description of specific actions, and no analysis of lessons learned or best practices in nursing homes during the pandemic. We then retrieved the full-text articles of the 167 remaining records, of which 15 met our inclusion criteria. Figure 1 summarizes the study selection process in a flowchart, including the main reasons for study exclusion.

### 3.2. Description of Included Studies

Five studies (33%) had a cross-sectional design, and another five (33%) were recommendations.

Regarding the study population, the studies included between one and 75 facilities, meaning the sample was unevenly distributed.

Together, the studies included 7379 residents and 2598 staff members, spread over 99 care homes. Two studies had no participants (staff or residents) [15,16] and a third study interviewed experts in the field [17]. The sample size varied widely between studies, with the regional studies of Luzón et al. [18] and Vijh et al. [14] having the largest numbers of participants.

Regarding geographical distribution, five studies took place in the USA, three in Spain, two each in Brazil and Canada, and one each in Saudi Arabia, France and Japan.

All included articles were related to the COVID-19 pandemic. We found no studies that examined lessons learned from previous epidemics, such as SARS-CoV or MERS-CoV.

### 3.3. Identification and Classification of Lessons Learned According to IDEF Methodology

**Figure 2:**
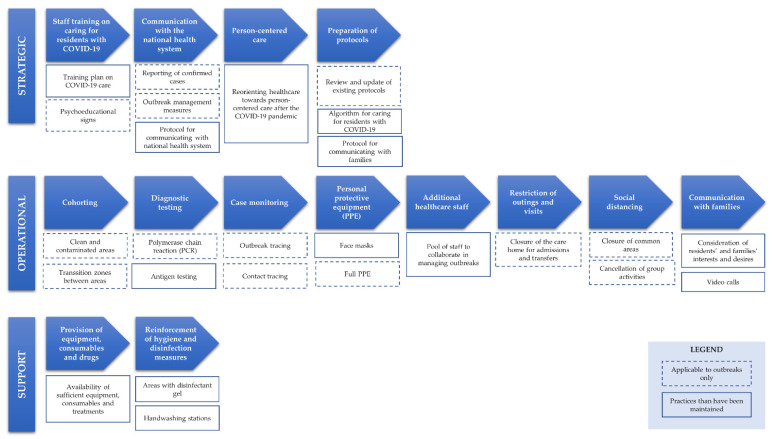
Good practices according to the IDEF model.

We identified 14 good practices and 26 specific actions from the COVID-19 pandemic management in care homes (Figure 2). According to the IDEF model, four good practices (25.6%) and nine actions (34.6%) corresponded to strategic processes, eight good practices (57.1%) and 14 actions (53.9%) were operational processes, and two good practices (14.3%) and three actions (11.5%) referred to support processes. Of the 26 practices analyzed, half (*n* = 13, 50%) were only applicable in an outbreak situation, and the other half (*n* = 13, 50%) had a broader scope that justified their maintenance beyond the pandemic as part of the core activity of the health care facility.

The strategic processes consisted of training staff to care for residents with COVID-19 through training activities in the care facilities [16]; posting educational signs explaining measures for reducing contamination [18,19,20]; establishing mechanisms for communicating with, and providing up-to-date information to, national health systems, based on electronic information systems for reporting cases, and coding medicines used for treating COVID-19 [14,20,21]; reorienting the center’s strategy by adopting a patient-centered care approach [21,22]; reviewing and updating existing protocols and creating a shared algorithm for managing residents with COVID-10 [18,20].

Within the strategic processes, the good practices maintained beyond the outbreak situation included the establishment of new organizational and care protocols and the implementation or strengthening of actions aimed at providing person-centered care [21,22].

Three practices were the most widespread in the framework of the operational processes: systematic polymerase chain reaction (PCR) testing of residents and professionals with symptoms of COVID as a mechanism for detecting positive cases and controlling infection [14,16,17,19,20,21,23,24,25,26,27]; the use of personal protective equipment (PPE) by facility personnel and masks by residents [14,15,16,17,19,20,21,23,24,25,26,27]; and restriction of outings and external visits [14,15,17,19,21,22,23,24,25,26,27,28]. Other practices commonly used included: case monitoring for the outbreak and contact tracing of COVID-19-positive individuals [14,15,19,20,21,23,25,26,27], cohort segmentation by identifying and monitoring transition zones regarding cleaning between contaminated area and other areas [14,16,18,20,21,25,26,28], and social distancing measures that included closing common areas (e.g., dining halls and classrooms) and canceling group activities [14,15,19,21,24,25,27,28]. Additional measures, reported to a lesser extent in the studies, were the establishment of new communication mechanisms with the residents’ families (recording their needs and setting up video calls between residents and their families) [21,24] and the incorporation of new reinforcement personnel to the center for crisis management [14,20,21]. Most of the best practices and specific actions included in the operational processes applied to the outbreak situation. However, using antigen testing for screening and case detection in the presence of symptoms of COVID-19 (or other contagious respiratory diseases) and alternative means of communication with residents’ families are practices that could be maintained after the crisis to improve the quality of care in nursing homes.

The practices that we classified as support processes were the provision of PPE, consumables, and treatments for use by staff and residents [14,20], and the provision of medicines by the national health system [18]. Another noteworthy support process was the reinforcement of hygiene measures and disinfection of the different areas of the care homes [14,20,24]. In this sense, the COVID-19 pandemic prompted the reinforcement of hygiene and disinfection practices [14,15,18,21], including the widespread use of additional hand hygiene stations in all rooms of care homes [28]. These measures are likely to be maintained as part of the facilities’ daily routine.

**Figure 3:**
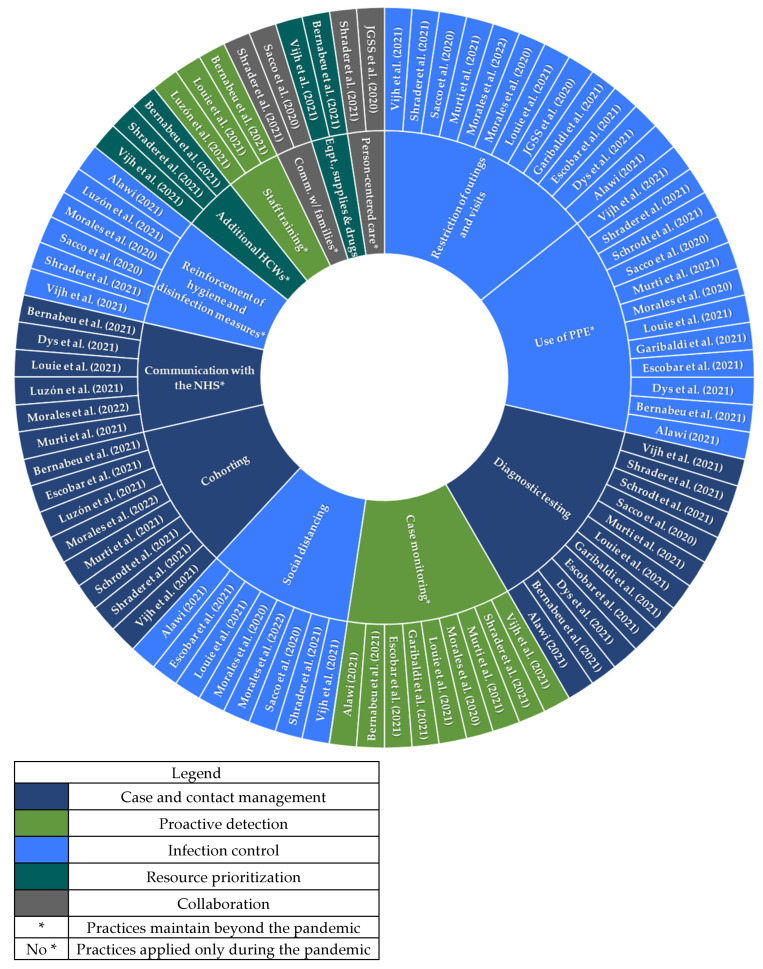
Frequency of good practices according to their categories. Comm.: Communication; Eqpt.: Equipment; HCWs: healthcare workers; JGSS: Japan Geriatrics Society Subcommittee; NHS: national health system; PPE: Personal protective equipment; w/: with. * Practices maintain beyond the pandemic. Those practices not marked with an asterisk apply only during the pandemic. Refs. [14,15,16,17,18,19,20,21,22,23,24,25,26,27,28].

Figure 3 shows the good practices that were described in at least two independent studies. In accordance with the proposal of Vijh et al. [14], the most common category of practices was infection control (80% use of PPE, 80% restriction of visits, 53% social distancing and 40% reinforcement of hygiene measures). In the category of case and contact management, 73% of studies included PCR testing, 53% cohorts of residents and staff, and 40% implemented mechanisms of communication with the national health system. Fewer practices belonged in the categories of collaboration (60% case monitoring, 13% person-centered care and 13% information and communication with families), resource prioritization (20% strengthening of human resources with incorporation of new staff, 13% provision of equipment and consumables) and proactive detection (13% specific professional training for handling residents with COVID-19, and hygiene and protective measures).

### 3.4. Narrative Synthesis of Studies about Lessons Learned in Care Homes during the COVID-19 Pandemic

The following describes the good practices identified in the literature review according to the classification proposed, based on the IDEF methodology.

#### 3.4.1. Good Practices Incorporated into the Strategic Processes of Care Homes during Pandemic

Concerning the strategic processes, the gerontological care centers carried out training activities aimed at the center’s professionals for the correct care of patients with COVID-19. According to the experience of Luzón et al. (2021) [18], Bernabeu-Wittel et al. (2021) [20], and Morales et al. (2022) [28] in a total of 81 Spanish centers, this training emphasized the management of the pathology and the actions to follow according to the referred symptomatology or the signs observed in the older adult. In addition, some centers, such as those in the study by Alawi (2021) [19] in Saudi Arabia, described the provision of educational signage in the facilities, consisting of informative posters reminding people of the need to wear masks, wash hands regularly, and correctly put on PPE.

Up to six studies included the reporting of confirmed cases to the National Health System [17,18,20,26,27,28]. According to Louie et al. (2021) [27], this notification was carried out daily and via telephone. Along the lines of outbreak management, the US studies by Schrodt et al. (2021) [16] and Shrader et al. (2021) [21] and the Canadian study by Vijh et al. (2021) [14] incorporated among their good practices the electronic admission of infected users, the epidemiological study to trace the origin of outbreaks and their evolution, the reporting of cases and their symptoms, and the record derived from the daily monitoring of those infected by the virus.

In Japan, during the COVID-19 pandemic, the Japan Geriatrics Society et al. (2020) [22] established the priority objective to promote shared decision-making and a care practice based on the principles of person-centered care. This methodology places the older adults at the center’s backbone, defining the care provided following their tastes, preferences, and wishes to facilitate the continuity of their life project and history.

Finally, regarding strategic processes, authors such as Bernabeu-Wittel et al. (2021) described the definition of protocols and algorithms for correctly managing COVID-19 [20].

#### 3.4.2. Good Practices Incorporated into Care Home Operational Processes during Pandemic

Regarding operational processes, one of the most frequent practices among the studies reviewed was the performance of mass PCR tests on all users of the centers [14,16,17,19,20,22,23,25,26,27]. In the same line of action, Shrader et al. (2021) [21] contemplated antigen testing of users and professionals regardless of whether or not they had symptoms compatible with COVID-19. Likewise, the use of masks and full PPEs was present in most studies [14,16,17,19,20,21,23,24,25,26,27,28].

Most of the studies incorporated, as a good practice, the spatial and functional delimitation of a clean and a contaminated area, placing users with SAR-CoV-2 infection in a different location from those not infected [14,16,18,20,21,25,26,27]. This solution was possible by redefining the usefulness and functionality of the center’s facilities by redistributing them into differentiated and properly marked areas. In this sense, Dys et al. (2021) [17] proposed the creation of a transition zone to adequately manage the transition from the contaminated area to the clean one.

Along the lines of minimizing the spread of the virus, most studies included social distancing measures [14,15,19,21,24,25,27,28]. In this regard, Sacco et al. (2020) [24] described the temporary closure of the center’s common areas. In Brazil, Garibaldi et al. (2021) [23] reported the cancelation of group activities, while Morales et al. (2020) [15] opted to reduce group therapy sessions.

Bernabeu et al. (2021), Shrader et al. (2021) [21], and Vijh et al., 2021 [14] highlighted the need for more healthcare professionals as a result of overwhelmed facility response capacity and to mitigate the adverse effects of the disease.

Regarding the relationship between users and their families, the Japan Geriatrics Society et al. (2020) [22], in their commitment to the patient-centered care paradigm, proposed as a recommended practice the recording of interests and wishes by users and their families. Along the same lines, Shrader et al. (2021) [21] incorporated video calls into the center’s daily routine to facilitate the contact of older adults with their family and social contexts.

#### 3.4.3. Good Practices Incorporated into Care Home Support Processes during Pandemic

Regarding support processes, Vijh et al. (2021) [14] and Bernabeu-Wittel et al. (2021) [20] identified the provision of equipment, consumables, and treatments as an essential process for quality care in nursing homes.

Finally, Sacco et al. (2020) [24] described the installation of disinfectant gel dispensers in all facility rooms and hand-washing areas as supporting practices for safe resident care.

## 4. Discussion

The population most affected by the COVID-19 pandemic, in terms of severity of illness and mortality, has been older people living in care homes [29]. Traditionally, care home residents have been largely disregarded in the health and social care sectors, and members of this population may face the additional problem of social stigma [30]. The COVID-19 pandemic aggravated these vulnerabilities, worsening its impact on care homes [31].

Lack of knowledge and the rapid spread of the virus, together with the unpreparedness of health systems in all parts of the world to deal with a health crisis of this magnitude, compelled care homes to improvise their own measures. All systems entered an operational mode of trial and error [32]. Some decisions and actions were undoubtedly correct, but not all. After the acute phase of the crisis, it is crucial to retrospectively analyze and reflect on what worked and what did not and identify new practices that are here to stay. The objective of this review was to identify the lessons learned from the management of the COVID-19 pandemic and other similar situations in care homes. The fact that we found no publications examining good practices adopted in previous recent epidemics (SARS-CoV or MERS-CoV) highlights the need for this reflection as a strategy to prepare health systems and long-term care facilities for future health crises of this nature.

Most practices for the successful management of the COVID-19 pandemic in care homes related to detecting and controlling positive cases and adopting protective and hygiene measures. Key organizational and structural measures included PCR testing, separating residents into different areas according to their clinical status, and using PPE.

In this review, we aimed to go one step further and categorize the lessons learned using the IDEF methodology, which organizes processes into functions and contributes to care quality and reduced variability [13]. We also adopted a forward-looking perspective of continual improvement by identifying practices that can be maintained, such as the use of video call technology for helping residents to keep in touch with their families and loves ones [21,24]. Looking beyond the pandemic, video calls could help to increase the quantity and quality of residents’ relationships with their families [33] and could even reduce agitation in residents with dementia [34]. The results of our review showed a general lack of focus on human needs. Most studies did not consider the experiences of residents, families, or staff during the pandemic. Although more than a third of the included publications included samples of residents and staff, both groups were considered agents and recipients of the contagion, but not as people with specific emotional and psychological needs. Van der Roest et al. [35] observed that the measures taken against COVID-19 in a long-term care facility in the Netherlands seriously affected residents’ well-being, increasing levels of loneliness and depression, and exacerbating behavioral and mood disorders. In this sense, adopting a person-centered care model is essential in care homes, more so in critical situations such as that caused by the COVID-19 pandemic [36]. This approach places residents’ tastes and preferences at the center of the care process [37], to guarantee excellent care based on the highest quality standards. 

Similarly, it is surprising that no included articles considered the perspective of the residents’ families. Avidor et al. [38] gave voice to this group of people, who indicated that the measures taken against COVID-19 had produced a feeling of rupture, in terms of separation from their family members, discontinuity of care, and dissatisfaction with, and distrust in, the facility. In addition, family members highlighted the need for facilities to respect their preferences and involve them in decision-making regarding the care of their loved ones and expressed a feeling of ambivalence caused by the combination of worry and distress with satisfaction and composure. Tretteteig et al. [39] identified the ethical dilemmas felt by loved ones when weighing the risk of contracting COVID-19 or passing it on while visiting relatives against the consequences of not visiting them. COVID-19 has made it more difficult for care homes to manage grief and provide dignified end-of-life care [40].

In the publications included in our review, the most common measure aimed at staff was training. However, the pandemic also had a strong impact on staff well-being, escalating levels of stress and worry [31]. One lesson learned from the COVID-19 pandemic concerns the need to implement measures aimed at strengthening the resilience of staff and preserving their well-being, to guarantee the proper functioning of long-term care facilities [41,42].

To the best of our knowledge, ours is the first systematic review to compile lessons learned from the pandemic in care homes. This reflection exercise is a first step towards consolidating the key measures to help long-term care facilities recover after the COVID-19 era. However, our review has some limitations. Most of the studies were observational and went no further than describing an experience. In some cases, the articles did not describe good practices in detail, which limited the depth of the narrative synthesis of the studies. Given the low quality of the studies, we were unable to determine the strength of the different recommendations.

## 5. Conclusions

The results of our review summarize the most common measures adopted to manage the COVID-19 pandemic in a context of increased vulnerability and highlight the deficiencies that must be addressed. Our findings also offer a vision of continued improvement by distinguishing practices specifically aimed at controlling the outbreak from those that could be useful in the daily activities of care homes. Future research should operationalize these good practices and evaluate their cost-effectiveness.

## Figures and Tables

**Table 1:** Search strategies for each database.

Database	Search Strategy	Results
Science Direct	(learnings [Title/Abstract]) AND (long-term care [Title/Abstract]) AND ((SARS [Title/Abstract]) OR (MERS [Title/Abstract])) NOT (hospital [Title/Abstract])	93
	(lessons [Title/Abstract]) AND (long-term care [Title/Abstract])) AND ((SARS [Title/Abstract]) OR (MERS [Title/Abstract])) NOT (hospital [Title/Abstract])	44
	((positive learnings [Title/Abstract]) OR (positive lessons [Title/Abstract])) AND ((nursing homes [Title/Abstract])) OR (long-term care [Title/Abstract])) AND ((SARS [Title/Abstract]) OR (MERS [Title/Abstract])) NOT (hospital [Title/Abstract])	829
	TOTAL	966
ProQuest	ti (learnings) AND ti (nursing homes) OR ti (long-term care) AND ti (SARS) OR ti (MERS) NOT ti (hospital)	700
	ti (lessons) AND ti (nursing homes) OR ti (long-term care) AND ti (SARS) OR ti (MERS) NOT ti (hospital)	694
	ti (positive learnings) OR ti (positive lessons) AND ti (nursing homes) OR ti (long-term care) AND ti (SARS) OR ti (MERS) NOT ti (hospital)	878
	TOTAL	2272
PubMed	(learnings [Title/Abstract]) AND ((nursing homes [Title/Abstract])) OR (long-term care [Title/Abstract])) AND ((SARS [Title/Abstract]) OR (MERS [Title/Abstract])) NOT (hospital [Title/Abstract])	252
	(lessons [Title/Abstract]) AND ((nursing homes [Title/Abstract])) OR (long-term care [Title/Abstract])) AND ((SARS [Title/Abstract]) OR (MERS [Title/Abstract])) NOT (hospital [Title/Abstract])	252
	((positive learnings [Title/Abstract]) OR (positive lessons [Title/Abstract])) AND ((nursing homes [Title/Abstract])) OR (long-term care [Title/Abstract])) AND ((SARS [Title/Abstract]) OR (MERS [Title/Abstract])) NOT (hospital [Title/Abstract])	212
	TOTAL	716
Scopus	(TITLE-ABS-KEY (learnings) AND TITLE-ABS-KEY (nursing AND homes) OR TITLE-ABS-KEY (long-term AND care) AND TITLE-ABS-KEY (sars) OR TITLE-ABS-KEY (mers) AND NOT TITLE-ABS-KEY (hospital))	49
	(TITLE-ABS-KEY (lessons) AND TITLE-ABS-KEY (nursing AND homes) OR TITLE-ABS-KEY (long-term AND care) AND TITLE-ABS-KEY (sars) OR TITLE-ABS-KEY (mers) AND NOT TITLE-ABS-KEY (hospital))	47
	(TITLE-ABS-KEY (positive AND learnings) OR TITLE-ABS-KEY (positive AND lessons) AND TITLE-ABS-KEY (nursing AND homes) OR TITLE-ABS-KEY (long-term AND care) AND TITLE-ABS-KEY (sars) OR TITLE-ABS-KEY (mers) AND NOT TITLE-ABS-KEY (hospital))	11
	TOTAL	101
Embase	((learnings:ab,ti AND ‘nursing homes’:ab,ti OR ‘long-term care’:ab,ti) AND sars:ab,ti OR mers:ab,ti) NOT hospital:ab,ti	824
	((lessons:ab,ti AND ‘nursing homes’:ab,ti OR ‘long-term care’:ab,ti) AND sars:ab,ti OR mers:ab,ti) NOT hospital:ab,ti	824
	((‘positive learnings’:ab,ti OR ‘positive lessons’:ab,ti OR ‘nursing homes’:ab,ti OR ‘long-term care’:ab,ti) AND sars:ab,ti OR mers:ab,ti) NOT hospital:ab,ti	824
	TOTAL	2472
	TOTAL	6527

## Data Availability

The data supporting reported results are available from the corresponding author, I.C., upon reasonable request.

## Supplementary Materials

The following supporting information can be downloaded at: https://www.mdpi.com/article/10.3390/ijerph192416919/s1, Table S1: Main findings and features of the studies reviewed. Refs. [14,15,16,17,18,19,20,21,22,23,24,25,26,27,28] are cited in the supplementary files.

## References

[1] Palacios Cruz M., Santos E., Velázquez Cervantes M.A., León Juárez M. (2021). COVID-19, una emergencia de salud pública mundial [COVID-19, a worldwide public health emergency]. Rev. Clin. Esp..

[2] Institut National d’Études Démographiques (INED) [National Institute for Demographic Studies] Demography of COVID-19 Deaths. https://covid-19-apport-demographie.site.ined.fr/en/research-projects/demography-of-covid-19-deaths.

[3] Bonanad C., García-Blas S., Tarazona-Santabalbina F.J., Díez-Villanueva P., Ayesta A., Sanchis Forés J., Vidán-Austiz M.T., Formiga F., Ariza-Solé A., Martínez-Sellés M. (2020). Coronavirus: La emergencia geriátrica de 2020. Documento conjunto de la Sección de Cardiología Geriátrica de la Sociedad Española de Cardiología y la Sociedad Española de Geriatría y Gerontología [Coronavirus: The Geriatric Emergency of 2020. Joint Document of the Section on Geriatric Cardiology of the Spanish Society of Cardiology and the Spanish Society of Geriatrics and Gerontology]. Rev. Clin. Esp..

[4] Huang C., Wang Y., Li X., Ren L., Zhao J., Hu Y. (2020). Clinical features of patients infected with 2019 novel coronavirus in Wuhan, China. Lancet.

[5] Zhou F., Yu T., Du R., Fan G., Liu Y., Liu Z., Xiang J., Wang Y., Song B., Gu X. (2020). Clinical course and risk factors for mortality of adult inpatients with COVID-19 in Wuhan, China: A retrospective cohort study. Lancet.

[6] Pan American Health Organization (PAHO) People over 60 Have Been Hardest Hit by COVID-19 in the Americas. https://www.paho.org/en/news/30-9-2020-people-over-60-have-been-hardest-hit-covid-19-americas.

[7] Lithander F.E., Neumann S., Tenison E., Lloyd K., Welsh T.J., Rodrigues J.C., Higgins J.P., Scourfield L., Christensen H., Haunton V.J. (2020). COVID-19 in older people: A rapid clinical review. Age Aging.

[8] Zunzunegui M.A. (2022). COVID-19 en centros residenciales de personas mayores: La equidad será necesaria para evitar nuevas catástrofes [COVID-19 in care homes: Equity will be needed to avoid new catastrophes]. Gac. Sanit..

[9] Vaqué Rafart J. (2005). Síndrome respiratorio agudo grave (SARS). An. Pediatr..

[10] Bratanich A. (2015). MERS-CoV: Transmisión y el papel de nuevas especies hospederas [MERS-CoV, transmisión and the role of new host species]. Rev. Argent. Microbiol..

[11] Page M.J., McKenzie J.E., Bossuyt P.M., Boutron I., Hoffmann T.C., Mulrow C.D., Shamseer L., Tetzlaff J.M., Akl E.A., Brennan S.E. (2021). The PRISMA 2020 statement: An updated guideline for reporting systematic reviews. BMJ.

[12] Munn Z., Stern C., Aromataris E., Lockwood C., Jordan Z. (2018). What kind of systematic review should I conduct? A proposed typology and guidance for systematic reviewers in the medical and health sciences. BMC Med. Res. Methodol..

[13] Saturno P.J. (2008). Diseño de Procesos. Método IDEFØ. Mapa de Procesos de una Organización. Manual del Máster en Gestión de la Calidad en Los Servicios de Salud. Módulo 5: Métodos y Herramientas Para el Diseño de la Calidad. Protocolización de Actividades Clínicas y Diseño de Procesos. Unidad Temática 30.

[14] Vijh R., Prairie J., Otterstatter M., Hu Y., Hayden A., Yau B., Daly P., Lysyshyn M., McKee G., Harding J. (2021). Evaluation of a multisectoral intervention to mitigate the risk of severe acute respiratory coronavirus virus 2 (SARS-CoV-2) transmission in long-term care facilities. Infect. Control Hosp. Epidemiol..

[15] Morales E.N., Viana L.G., Resende L.M., Vasconcellos L.S., Moura A.S., Menezes A., Mansano N.H., Rabelo R. (2020). COVID-19 in long-term care facilities for the elderly: Laboratory screening and disease dissemination prevention strategies. Cienc. Saude Coletiva.

[16] Schrodt C.A., Malenfant J.H., Hunter J.C., Slifka K.J., Campbell A., Stone N., Whitehouse E.R., Wittry B., Christensen B., Barnes J.R. (2021). Investigation of a Suspect Severe Acute Respiratory Syndrome Coronavirus-2 and Influenza A Mixed Outbreak: Lessons Learned for Long-Term Care Facilities Nationwide. Clin. Infect. Dis..

[17] Dys S., Winfree J., Carder P., Zimmerman S., Thomas K.S. (2021). Coronavirus Disease 2019 Regulatory Response in United States-Assisted Living Communities: Lessons Learned. Front. Public Health.

[18] Luzón Oliver L., Molina Pérez de Los Cobos E., Novoa Jurado A., Pérez Martínez E., Martínez Monreal D., Grupo CORECAAS (2021). La seguridad del paciente en las residencias sociosanitarias. La experiencia de la Comunidad Autónoma de la Región de Murcia [Patient safety in nursing homes. The experience of the Autonomous Community of the Region of Murcia]. Aten. Primaria.

[19] Alawi M.M. (2021). Successful management of COVID-19 outbreak in a long-term care facility in Jeddah, Saudi Arabia: Epidemiology, challenges for prevention and adaptive management strategies. J. Infect. Public Health.

[20] Bernabeu-Wittel M., Ternero-Vega J.E., Nieto-Martín M.D., Moreno-Gaviño L., Conde-Guzmán C., Delgado-Cuesta J., Rincón-Gómez M., Díaz-Jiménez P., Giménez-Miranda L., Lomas-Cabezas J.M. (2021). Effectiveness of a On-site Medicalization Program for Nursing Homes With COVID-19 Outbreaks. J. Gerontol. A Biol. Med. Sci..

[21] Shrader C.D., Assadzandi S., Pilkerton C.S., Ashcraft A.M. (2021). Responding to a COVID-19 Outbreak at a Long-Term Care Facility. J. Appl. Gerontol..

[22] Kuzuya M., Aita K., Katayama Y., Katsuya T., Nishikawa M., Hirahara S., Miura H., Yanagawa M., Arai H., Japan Geriatrics Society Subcommittee on End-of-Life Issues and New Coronavirus Countermeasure Team (2020). The Japan Geriatrics Society consensus statement “recommendations for older persons to receive the best medical and long-term care during the COVID-19 outbreak—Considering the timing of advance care planning implementation”. Geriatr. Gerontol. Int..

[23] Garibaldi P.M., Ferreira N.N., Moraes G.R., Mourac J.C., Espósito D.L., Volpe G.J., Caladoe R.T., Fonsecad B.A., Borges M.C. (2021). Efficacy of COVID-19 outbreak management in a skilled nursing facility based on serial testing for early detection and control. Braz. J. Infect. Dis..

[24] Sacco G., Foucault G., Briere O., Annweiler C. (2020). COVID-19 in seniors: Findings and lessons from mass screening in a nursing home. Maturitas.

[25] Escobar D.J., Lanzi M., Saberi P., Love R., Linkin D.R., Kelly J.J., Jhala D., Amorosa V., Hofmann M., Doyon J.B. (2021). Mitigation of a Coronavirus Disease 2019 Outbreak in a Nursing Home Through Serial Testing of Residents and Staff. Clin. Infect. Dis..

[26] Murti M., Goetz M., Saunders A., Sunil V., Guthrie J.L., Eshaghi A., Zittermann S., Teatero S., Fittipaldi N., Rilkoff H. (2021). Investigation of a severe SARS-CoV-2 outbreak in a long-term care home early in the pandemic. CMAJ.

[27] Louie J.K., Scott H.M., DuBois A., Sturtz N., Lu W., Stoltey J., Masinde G., Cohen S., Sachdev D., Philip S. (2021). Lessons From Mass-Testing for Coronavirus Disease 2019 in Long-Term Care Facilities for the elderly in San Francisco. Clin. Infect. Dis..

[28] Morales Viera A., Rivas Rodriguez R., Otero Aguilar P., Briones Pérez de Blanca E. (2022). Epidemiology of COVID-19 among health personnel in long-term care centers in Seville. Rev. Clin. Esp..

[29] Thompson D.C., Barbu M.G., Beiu C., Popa L.G., Mihai M.M., Berteanu M., Popescu M.N. (2020). The Impact of COVID-19 Pandemic on Long-Term Care Facilities Worldwide: An Overview on International Issues. BioMed Res. Int..

[30] Zimmerman S., Dobbs D., Roth E.G., Goldman S., Peeples A.D., Wallace B. (2016). Promoting and Protecting Against Stigma in Assisted Living and Nursing Homes. Gerontologist.

[31] Seshadri S., Concannon C., Woods J.A., McCullough K.M., Dumyati G.K. (2021). “It’s like fighting a war with rocks”: Nursing home healthcare workers’ experiences during the COVID-19 pandemic. Infect. Control Hosp. Epidemiol..

[32] Rassouli M., Ashrafizadeh H., Shirinabadi Farahani A., Akbari M.E. (2020). COVID-19 Management in Iran as One of the Most Affected Countries in the World: Advantages and Weaknesses. Front. Public Health.

[33] Hado E., Friss Feinberg L. (2020). Amid the COVID-19 Pandemic, Meaningful Communication between Family Caregivers and Residents of Long-Term Care Facilities is Imperative. J. Aging Soc. Policy.

[34] Van der Ploeg E.S., Eppingstall B., O’Connor D.W. (2016). Internet video chat (Skype) family conversations as a treatment of agitation in nursing home residents with dementia. Int. Psychogeriatr..

[35] Van der Roest H.G., Prins M., van der Velden C., Steinmetz S., Stolte E., van Tilburg T.G., de Vries D.H. (2020). The Impact of COVID-19 Measures on Well-Being of Older Long-Term Care Facility Residents in the Netherlands. J. Am. Med. Dir. Assoc..

[36] Díaz-Veiga P., Sanchoa M., García A., Rivas E., Abad E., Suárez N., Mondragón G., Buiza C., Orbegozo A., Yangua J. (2014). Efectos del Modelo de Atención Centrado en la Persona en la calidad de vida de personas con deterioro cognitivo de centros gerontológicos [Effects from the Person Centered-Care Model on quality of life of cognitive impaired persons from gerontological centers]. Rev. Esp. Geriatr. Gerontol..

[37] Pascual López J.A., Gil Pérez T., Sánchez J.A., Menárguez Puche J.F. (2020). Cuestionarios de atención centrada en la persona en atención primaria. Una revisión sistemática [Questionnaires of person centered care in primary care. A systematic review]. Aten. Primaria.

[38] Avidor S., Ayalon L. (2022). “I Didn’t Meet My Mother; I Saw My Mother”: The Challenges Facing Long-Term Care Residents and Their Families in the Age of COVID-19. J. Appl. Gerontol..

[39] Tretteteig S., Eriksen S., Hillestad A.H., Julnes S.G., Lichtwarck B., Nilsen A., Rokstad A.M. (2022). The Experience of Relatives of Nursing Home Residents with COVID-19: A Qualitative Study. Nurs. Res. Rev..

[40] Vellani S., Boscart V., Escrig-Pinol A., Cumal A., Krassikova A., Sidani S., Zheng N., Yeung L., McGilton K.S. (2021). Complexity of Nurse Practitioners’ Role in Facilitating a Dignified Death for Long-Term Care Home Residents during the COVID-19 Pandemic. J. Pers. Med..

[41] Bodenheimer T., Sinsky C. (2014). From triple to quadruple aim: Care of the patient requires care of the provider. Ann. Fam. Med..

[42] Ruiz-Frutos C., Gómez-Salgado J. (2021). Efectos de la pandemia por COVID-19 en la salud mental de la población trabajadora [Effects of the Covid-19 Pandemic on Workers’ Mental Health]. Arch. Prev. Riesgos Labor..

