# Supplementary material for: Lessons Learned from the COVID-19 Pandemic in Nursing Homes: A Systematic Review
Source: Int J Environ Res Public Health. 2022 Dec 16;19(24):16919. doi: 10.3390/ijerph192416919 (PMC9779143; doi:10.3390/ijerph192416919)
Supplement: Supplementary file 1 [file ijerph-19-16919-s001.zip › ijerph-2057408-supplementary.pdf]

International Journal of
Environmental Research and Public Health

MDPI

Table S1. Main findings and features of the studies reviewed

Author (year)	Title	Objectives	Study design	Sample size	Main findings	Discussion and conclusion
Alawi (2021) [19]	Successful management of COVID-19 outbreak in a long-term care facility in Jeddah, Saudi Arabia: Epidemiology, challenges for prevention and adaptive management strategies	To report the case of a successful management of a COVID-19 outbreak in a long-term care facility in Western Saudi Arabia	Case study	1 care facility with 100 beds for the care of older adults and 6 beds in a cardiovascular care unit	• Closure of the facility for admissions and transfers<br>• Contact tracing<br>• Screening of all residents<br>• Implementation of social distancing and posting of educational signs<br>• Reporting to infection control team and clinical staff of symptoms among residents and staff<br>• Reinforcement of environmental cleaning<br>• Compliance with use of PPE<br>• Limitation of the use of the institution's bus<br>• Monitoring of cleaning and disinfection practices<br>• Reduction of the risk of equipment cross-transmission with a cleaning log	This study highlights the need for and importance of continuous training and sensitizing healthcare personnel on best practices for self-protection and patient protection. It also emphasizes the role of psychological support as a means of facilitating staff adherence to safety and prevention guidelines and their self-efficacy.
Bernabeu-Wittel et al. (2021) [20]	Effectiveness of a On-site Medicalization Program for Nursing Homes With COVID-19 Outbreaks	To describe an innovative, coordinated, on-site medicalization program, which was carried out in response to a sizeable COVID-19 outbreak, with the aim of ensuring medically rigorous but also humanistic care to residents with COVID-19 in their natural environment	Cross-sectional and cohort study	777 older adults from 4 nursing homes in the province of Seville (Spain)	• Clean room' for clinical work<br>• Locker room with PPE<br>• Universal SARS-CoV-2 testing<br>• 'Clean area' and 'contaminated area' with rooms and common spaces<br>• Specific training in the care of COVID-19 patients<br>• Epidemiological survey to trace outbreaks' origin and evolution<br>• Electronic admission of residents with infection<br>• Provision of equipment, expendable materials and drugs<br>• Provision of healthcare workers<br>• Clinical management and treatment algorithm and communication protocol	The coordinated on-site medicalization program implemented in four nursing homes with outbreaks of COVID-19 contributed to an improvement in the survival rate or quality of palliative care and a reduction in hospital referrals. Beyond rigorous care, the program also improved the humaneness and gentleness of care for residents.
Dys et al. (2021) [17]	Coronavirus Disease 2019 Regulatory Response in United States-Assisted Living Communities: Lessons Learned	To examine COVID-19 regulatory and policy response in U.S. assisted living and residential care settings, ultimately to discern lessons learned for the future	Qualitative study	42 staff members	• Policymakers are disconnected from and lack an understanding of the context<br>• Administrators were left to coordinate, communicate and implement constantly changing guidelines with little support<br>• Organizations faced limited knowledge of and disparate access to funding and resources	This work represents a first step along the lines of sharing knowledge and learning from the experiences of U.S. assisted living stakeholders in the aftermath of the COVID-19 pandemic for the subsequent development of rules and regulations that seek to impact positively

					• State-level regulatory requirements conflicted with COVID-19 guidelines, resulting in uncertainty about which rules to follow<br>• Operators struggled to balance public health priorities with promoting their residents' quality of life and well-being	the care offered in assisted living facilities. These policies' development involves considering input from all stakeholders involved in assisted living (from workers and clinicians to residents).
Escobar et al. (2021) [25]	Mitigation of a Coronavirus Disease 2019 Outbreak in a Nursing Home Through Serial Testing of Residents and Staff	To describe a COVID-19 outbreak in a nursing home in eastern Pennsylvania that was rapidly contained by using a universal testing strategy of all residents and nursing home staff	Case study Description of an experience	84 residents	• PCR testing with turnaround times of 3 to 24 hours<br>• No-visitor policy<br>• Symptom screening at entrance<br>• Closure of common areas<br>• Closure to new admissions<br>• Nebulizers switched to metered dose inhalers<br>• Testing based on symptoms<br>• Universal mask-wearing policy for staff<br>• New isolation unit<br>• Routine care provided in rooms<br>• Residents prohibited from travelling between units<br>• Implementation of mask-wearing for all residents<br>• Quarantine in rooms<br>• Eye shields for clinical staff<br>• Staff cohorted to specific units	This case report supports serial testing of residents and staff in short-time intervals (testing residents every 3-5 days). This testing strategy led to early isolation and cohorting, likely preventing additional transmission. Universal testing of staff allowed for the identification of positive pre-symptomatic staff members.
Garibaldi et al. (2021) [23]	Efficacy of COVID-19 outbreak management in a skilled nursing facility based on serial testing for early detection and control	To report a successful outbreak investigation and management, based on serial point-prevalence for early detection and control in a skilled nursing facility in a middle-sized city in São Paulo State, Brazil	Cross-sectional and cohort study	23 long-term residents and 26 healthcare workers	• Early identification of symptomatic and asymptomatic cases and contact tracing<br>• Staff training on the correct use of PPE<br>• Recommended use of masks<br>• Cancellation of visits and group activities	Optimal management of the COVID-19 outbreak requires: (1) appropriate testing of asymptomatic individuals, residents, and staff in long-term care and assisted living facilities; (2) continuous staff training and appropriate use of PPE; and (3) active surveillance and early isolation of residents who test positive for SARS-CoV-2 infections.
Japan Geriatrics Society et al. (2020) [22]	The Japan Geriatrics Society consensus statement "recommendations for older persons to receive the best medical and long-term care during the COVID-19 outbreak – considering the timing of	To announce the ethical recommendations of the Japan Geriatrics Society for medical and long-term care for older persons and emphasize the importance of conducting advance care planning at earlier stages	Case study Description of an experience		• Providing best medical and long-term care and promoting shared decision-making:<br>o The right to best medical and long-term care should be guaranteed<br>o Advance care planning should be promoted to ensure the right to best medical and long-term care until the end of life<br>o The end-of-life care desired by older people should be guaranteed	These recommendations from the Japan Geriatrics Society comprise ethical suggestions aimed at achieving a society in which older people can receive the best medical and long-term care they deserve, even during the COVID-19 pandemic. The recommendations do not refer

	advance care planning implementation”				• Concrete practice of advance care planning during the COVID-19 pandemic:<br>o Medical information must be shared with the resident and family, and active decision support is necessary<br>o It is necessary to ensure communication between the older person and family, and medical and long-term care workers<br>o An appropriate ventilator wearing/withdrawal approach is required in accordance with the guidelines<br>• Providing an appropriate medical and long-term care environment and supporting family and caregivers<br>o It is necessary to consider the wishes of patient and family to provide place for medical and long-term care<br>o It is necessary to establish an appropriate infection control support system for older persons and their families in the community<br>o It is necessary to provide appropriate care to families and caregivers<br>• Elimination of prejudice/discrimination against those involved in COVID-19<br>o Prejudice and discrimination against residents with COVID-19/family members and medical/care workers involved in treatment and infection control should be eliminated	to treatment or specific preventive measures for older persons against COVID-19. Rather, their purpose is to consider the timing of advance care planning implementation, not to mention the importance of its application.
Louie et al. (2021) [27]	Lessons From Mass-Testing for Coronavirus Disease 2019 in Long-Term Care Facilities for the elderly in San Francisco	To describe the results of surveillance, outbreak response, and control measures to prevent COVID-19 transmission in 4 San Francisco long-term care facilities early in the pandemic	Case study Description of an experience	431 residents in 4 long-term care facilities	• Active surveillance with daily calls to the skilled nursing facilities and appropriate use of PPE<br>• PCR testing in suspected cases<br>• An outbreak was defined as at least one confirmed case of COVID-19<br>• Tracing of close contacts after the identification of an outbreak<br>• Implementation of mass testing	The results suggest that symptom-based monitoring is ineffective for detecting COVID-19 among staff or elderly residents in hospice settings. Residents' cognitive impairment is a barrier to symptom identification. Recommended practice is, therefore, routine facility-wide testing to detect asymptomatic and pre-symptomatic COVID-19 early. Another measure to limit inter-facility transmission is the implementation of supportive and non-punitive policies for healthcare professionals

						infected with COVID-19 for home isolation.
Luzón et al. (2021) [18]	Patient safety in nursing homes. The experience of the Autonomous Community of the Region of Murcia	To describe the components and results of a model developed to control infections and intervention in SARS-CoV-2 outbreaks in nursing homes in the Autonomous Community of the Region of Murcia. To analyze the risks from the perspective of patient safety using Reason's Swiss cheese model of accident causation, to determine the underlying reasons for the impact of the pandemic in nursing homes and propose improvements	Case study<br>Description of an experience	63 nursing homes in the Autonomous Community of Murcia (Spain), with 4386 residents.	• Structure:<br>o Adaptation of physical spaces to safer care models<br>o Lower occupancy<br>o Individual rooms with their own bathrooms<br>o Well-ventilated buildings with open spaces<br>• Organization:<br>o Regional coordination capable of organizing, coordinating resources and systematizing interventions in nursing homes in a homogeneous and coordinated way, with a global vision<br>o Establishment of quality standards in the nursing homes, evaluated by the administration and that serve as a reference for the price scales in the publicly funded places<br>o Systematization of test orders and turnaround<br>o Provision of top-tier equipment<br>• Training:<br>o Training of nursing home staff in preventive measures, caring for frail patients, palliative care<br>o Certification of professional skills<br>o Professionalization of management teams<br>o Provision of protocols for diagnosing and treating residents<br>o Specific training on frailty<br>• Coordination:<br>o Creation of commissions involving all health actors<br>o Improved coordination between care levels between crises<br>o Improved coordination between nursing homes and primary care between crises<br>• Human resources:<br>o Improved ratios of professionals of all categories<br>o Improved working conditions and wages<br>• Material resources:<br>o Provision of the same resources available to patients in the community<br>• Care culture:<br>o Health and care training related to end of life, ageing, frailty and the limits of medicine<br>o Shared care planning with families and residents<br>• Information systems:	The pandemic has brought to light the weakness of the nursing home system and the lack of coordination between health and social care. It is necessary to implement changes that promote a more equitable care model, that better meets current needs, and that goes beyond an exclusively biomedical model to consider the preferences of residents and their families.

					○ Provision of an operating system for sharing of information between professionals in nursing homes and in the healthcare system<br>○ Creation of outbreak-specific forms in medical health records<br>○ Virtual hospitalization of residents treated in nursing homes<br>○ Proposal of access profiles for hospital software	
Morales et al. (2022) [28]	Epidemiology of COVID-19 among health personnel in long-term care centers in Seville	To analyze COVID-19 epidemiology among healthcare staff based in the Seville healthcare district (Spain) and evaluate its role in outbreaks in nursing homes	Cross-sectional and cohort study<br>Regional study	732 healthcare workers in 14 nursing homes	• Inclusion of daily tracking logs in information sources<br>• PCR testing<br>• Collection of variables: age, sex, professional category, confirmed close contact with COVID-19, hospitalization, death, presence of risk factors, symptoms and dates of symptom onset, isolation, declaration and test results	Transmission in the outbreaks could have been affected by delayed isolation of staff in COVID-19-positive nursing homes. Reinforcement of disease identification and staff isolation practices would have been essential to control the outbreak. Also, rapid implementation of prevention measures is needed for better infection control in nursing homes.
Morales et al. (2020) [15]	COVID-19 in long-term care facilities for the elderly: laboratory screening and disease dissemination prevention strategies	Strategies for researching this infection in residents and workers in long-term care facilities for the elderly using laboratory tests available in Brazil.	Case study	Not available	• Restriction of visits and control of access of staff and service providers, with the requirement of thorough and adequate handwashing or use of 70% alcohol gel<br>• Screening for the presence of flu-like symptoms (fever and respiratory symptoms) and change of clothes and shoes<br>• Distancing; greater separation between workers and older adults in non-care activities<br>• Reduction of group activities and collective meals<br>• Reinforcement of hygiene and cleaning measures<br>• Recommended mark use for all staff and residents in activities outside bedrooms<br>• Staff should avoid contact with other long-term care facilities or health services during the pandemic, particularly with confirmed COVID-19 cases	Long-term care facilities should be able to implement COVID-19 prevention strategies, based on the following principles: traditional disease control and prevention measures; removal of any worker with flu-like symptoms; immediate transfer of symptomatic residents to a healthcare facility with isolation units; PCR testing; and weekly screening of asymptomatic individuals with immunological tests.
Murti et al. (2021) [26]	Investigation of a severe SARS-CoV-2 outbreak in a long-term care home early in the pandemic	To report on the epidemiological data, infection prevention and control, laboratory testing and phylogenetic analysis of one of the earliest and largest COVID-19 outbreaks in an Ontario long-	Cross-sectional and cohort study	Care home with 65 residents and 67 staff members	• Expanded test indications<br>• Visitor policies<br>• Staff work locations<br>• Universal masking<br>• Strict outbreak definition and management	Lessons learned in a long-term care home were related to measures for early identification and prevention of symptoms, cases, and outbreaks. Control of transmission of infection involves active screening and universal masking of staff and

		term care home, and the impacts of progressive outbreak management measures that eventually influenced provincial guidance for long-term care outbreak management				mass testing of residents. Likewise, optimal staffing of professionals and PPE is a prerequisite for the SARS-CoV-2 outbreak management. In the long term, improvements in infrastructure, density, and staffing of long-term care facilities need to be rethought.
Sacco et al. (2020) [24]	COVID-19 in seniors: Findings and lessons from mass screening in a nursing home	To comprehensively describe symptoms and chronological aspects of the diffusion of the SARS-CoV-2 virus in a nursing home, among both residents and caregivers	Cross-sectional and cohort study	87 residents and 92 staff members	• Isolation in bedrooms<br>• No visitors, including families, allowed in the nursing home<br>• Walks organized for residents one by one<br>• Residents could receive their families by phone or video<br>• Mail and packages were stored for 24 hours before being delivered to residents<br>• Implementation of hygiene measures, including cleaning and disinfection of frequently touched surfaces<br>• Continual use of face masks<br>• Additional hand hygiene stations for staff	Mass screening in nursing homes is essential in the SARS-CoV-2 health crisis management, given the low symptomatic expression of COVID-19 in elderly residents and the high prevalence of asymptomatic forms in caregivers. It is advisable to prioritize residents with clinical signs of dyspnea, falls, anorexia, or altered consciousness. Isolation and visitor restriction measures are also essential to break the chain of contamination.
Schrodt et al. (2021) [16]	Investigation of a Suspect Severe Acute Respiratory Syndrome Coronavirus-2 and Influenza A Mixed Outbreak: Lessons Learned for Long-Term Care Facilities Nationwide	To describe an investigation of a suspected mixed influenza and SARS-CoV-2 outbreak to determine whether inconsistent cohort and infection control practices, along with non-specific laboratory testing, contributed to SARS-CoV-2 transmission.	Case study Description of an experience	102 residents and 123 staff members	• PCR testing<br>• Cohorting<br>• Use of PPE<br>• Communication with the health system<br>• Specific training	Lessons learned include familiarizing clinicians with the local epidemiology of influenza and SARS-CoV-2, performing antigen detection assays, and, if positive, confirming with PCR before making cohort decisions. Pooling of residents with suspected COVID-19 should only be performed after laboratory confirmation of infection. All residents with symptoms consistent with COVID-19 should be tested for SARS-CoV-2.
Shrader et al. (2021) [21]	Responding to a COVID-19 Outbreak at a Long-Term Care Facility	To describe an outbreak of COVID-19 in a long-term care facility in West	Case study	98 residents and 56 staff members	• Early testing<br>• Early isolation<br>• Daily availability of doctors<br>• Communication with the local health system<br>• Community-centered approach	Optimal management of a COVID-19 outbreak in a long-term care facility should include procedures for testing, infection control, treatment, and

		Virginia that was the epicenter of the state's pandemic.				communication with facility residents, staff, and family members.
Vijh et al. (2021) [14]	Evaluation of a multisectoral intervention to mitigate the risk of severe acute respiratory coronavirus virus 2 (SARS-CoV-2) transmission in long-term care facilities	To provide a descriptive overview of LTCF COVID-19 outbreaks in the Vancouver Costal Health region (British Columbia, Canada); to evaluate the effectiveness of the intervention (a bundle of outbreak control measures) in terms of reducing subsequent transmission among residents and staff; and to inform the ongoing public health approach to managing COVID-19 outbreaks in LTCFs	Before-and-after study without control group	7 LTCFs: 1144 residents and 1298 staff members	• Notification of all staff members for assessment of symptoms and linkage to tests<br>• Rigorous case follow-up, contact tracing and exclusion of high-risk contacts<br>• Listing of new cases and proactive follow-up of SARS-CoV-2 test results for all residents and staff tested the day before<br>• Daily monitoring of staff and residents to detect symptoms<br>• Low threshold for SARS-CoV-2 testing<br>• PPE precautions for all staff<br>• Contact and droplet precautions for confirmed, suspected or exposed cases of COVID-19<br>• Assessment, education, and ongoing support from a dedicated COVID-19 mobile IPAC team<br>• Closure of facility to all admissions or community discharges<br>• Restriction mobility of residents, with in-room dining<br>• Cohorting of staff to specific floors, wards, or units<br>• Cohorting of COVID-19 resident cases to specific floors, wards, units, or rooms<br>• Improved cleaning of the facility<br>• Proactive daily check-in with regional long-term care operation leads<br>• Deployment of necessary resources (i.e. additional staff) and PPE in a timely fashion<br>• Low barrier/preferential access to SARS-CoV-2 testing and rapid processing of test specimens<br>• These outbreak measures were implemented and maintained by using a team-based approach	An intervention based on four action blocks (case and contact management, proactive case detection, rigorous infection control practices, and prioritization and resource management) was effective in reducing COVID-19 transmission in long-term care facilities.

AL: assisted living; COVID-19: coronavirus disease 2019; LTC: long-term care; LTCF: long-term care facility; MP: medicalization program; PCR: polymerase chain reaction; PPE: personal protective equipment; SARS-CoV-2: severe acute respiratory syndrome coronavirus 2; SNF: skilled nursing facilities.
